# Psychological Stress among Students in Health-Related Fields during the COVID-19 Pandemic: Results of a Cross-Sectional Study at Selected Munich Universities

**DOI:** 10.3390/ijerph18126611

**Published:** 2021-06-19

**Authors:** Kristina Schröpfer, Nicole Schmidt, Sandra Kus, Clemens Koob, Michaela Coenen

**Affiliations:** 1 Institute for Medical Information Processing, Biometry, and Epidemiology—IBE, Chair of Public Health and Health Services Research, LMU Munich, 81377 Munich, Germany; kristina.schroepfer@ibe.med.uni-muenchen.de (K.S.); skus@ibe.med.uni-muenchen.de (S.K.); 2 Pettenkofer School of Public Health, 81377 Munich, Germany; 3 Department of Social Work, Catholic University of Applied Sciences, 81667 Munich, Germany; Nicole.Schmidt@ksh-m.de; 4 Gynecology Division, Department of Pediatrics, Obstetrics and Gynecology, University Hospitals of Geneva, 1205 Genève, Switzerland; 5 Department of Health and Nursing, Catholic University of Applied Sciences, 81667 Munich, Germany; Clemens.Koob@ksh-m.de

**Keywords:** coronavirus, COVID-19, mental health, psychological stress, students

## Abstract

The COVID-19 pandemic has been a challenging period of upheaval for higher education students. This study aims to assess the factors associated with psychological stress during the COVID-19 pandemic among a sample of students in health-related fields at Munich universities in Germany. Students (*n* = 623) from KSH Munich and LMU Munich completed an online cross-sectional survey. Information on demographics and academic and everyday difficulties due to the COVID-19 pandemic as well as data on physical and mental health were collected. Multivariable logistic regression analyses were performed to identify factors associated with the outcome. The prevalence for higher psychological stress was 44% among the study population. Factors associated with higher psychological stress were: lower overall life satisfaction (*p* < 0.0001), worsened health situation (*p* < 0.0001), lack of social support (*p* = 0.0301) and social interaction (*p* = 0.0115), worries about financial difficulties due to loss of income (*p* = 0.0134), stressful thoughts about a second wave (*p* < 0.0001), feeling unable to positively influence the situation (*p* = 0.0262) and study-related effects, such as perceived study burden (*p* = 0.0003) and likely delay in studies (*p* = 0.0178)). The COVID-19 pandemic is having a significant negative impact on the mental health of students in health-related fields. Proactive efforts to support the mental health and well-being of students are needed.

## 1. Introduction

The COVID-19 pandemic has led to a public health crisis worldwide [1]. According to the World Health Organization (WHO), as of 16 April 2021 there were close to 140 million confirmed cases of COVID-19 worldwide, with roughly 3 million reported deaths [2]. In addition to increased mortality rates, mental health issues have rapidly generated a public health burden [1]. The COVID-19 pandemic has had a huge impact on everyday life, and the tertiary education sector was severely affected by the pandemic [3]. To contain widespread transmission of the disease, authorities established measures with far-reaching consequences; in the higher education sector, universities in many parts of the world switched to online lectures and students’ lives changed drastically in a short period of time [4,5]. 

This transition to newly structured study courses and the pervasive uncertainty with the risk of prolonged study periods due to restructuring presented an important challenge for students [4]. In addition to teaching-related changes, loneliness due to social distancing is a further obstacle; being a student during lockdown poses a higher risk for loneliness [6]. Physical distancing measures as well as switching to online learning have resulted in isolation [7], which may cause anxiety and depression [8]. Young adults are one of the most vulnerable groups regarding the psychological consequences of the pandemic [9,10], although they are least likely to experience a severe or critical course when infected with the coronavirus [11]. Financial issues present an additional challenge, particularly among those who are self-sufficient or rely on relatives working in sectors that have been severely impacted by prolonged closures [12]. The cumulative burden of these stressors might have a significant impact on a student’s health and well-being. Several studies have shown that levels of stress, anxiety, loneliness, suicidal ideation and depressive symptoms have worsened compared to measures before the pandemic [9,13,14], and being a student has been identified as a risk factor associated with distress during the pandemic [15].

Students from the medical and health-related educational sector might be especially affected by the COVID-19 pandemic [7]. A study conducted on a population of German medical students reported high levels of distress associated with study-related concerns during the COVID-19 pandemic [16]. According to a French study, factors associated with psychological stress among university students were female gender, precariousness, history of psychiatric follow-up and social isolation [17]. Furthermore, Elmer et al. found in a cohort of undergraduate students that COVID-19-specific worry, isolation, lack of interaction and emotional support were associated with negative mental health [9]. On the other hand, a cross-sectional study of medical students in Japan characterized self-efficacy and self-esteem as predictive factors for lower levels of psychological distress [18].

Given the slow start of the vaccination campaign in most of the European countries [19] and no close end in sight for an unrestricted return to in-person teaching, it is important to identify COVID-19-related stressors that contribute to stress in this vulnerable population to be able to establish appropriate supportive structures and processes to prevent adverse long-term consequences. However, to our knowledge, up to now no comparable study has used such a wide variety of independent variables from different topics as our study when analyzing psychological stress among students during the COVID-19 pandemic. This study thus aims to identify factors associated with psychological stress during the COVID-19 pandemic among students in health-related fields.

## 2. Materials and Methods

### 2.1. Design

This cross-sectional study was conducted as an online survey and a collaborative effort of the Chair of Public Health and Health Services Research at the Institute for Medical Information Processing, Biometry, and Epidemiology (IBE) of LMU Munich and the Catholic University of Applied Sciences (KSH) Munich. The study protocol was approved by the responsible ethics committees of both universities. 

The study was performed in July 2020 in Munich, Bavaria, during the first lockdown due to the COVID-19 pandemic in Germany, which started on 22 March 2020. Measures were gradually eased by May, due to a lower COVID-19 incidence in Germany. During the summer months of July and August 2020, universities remained closed, while public locations reopened under hygiene restrictions such as masking and social distancing [20]. 

### 2.2. Participants

Eligible participants were students aged 18 years or older studying at one of the participating faculties which offer studies in medicine, nursing and other health-related fields, such as epidemiology, health care management, public health and social work sciences both on the bachelor’s and master’s levels. All participants agreed to the informed consent form before starting the online questionnaire.

### 2.3. Material

We established a self-administered questionnaire for this study. Wherever possible, we included standardized questionnaires and items of established questionnaires, respectively. Detailed information on the questionnaire used in this study, including all questions with response options, can be found in Table S1 of the Supplementary Material.

#### 2.3.1. Dependent Variable

Psychological stress associated with the COVID-19 pandemic and its restrictions as experienced by the students was used as the primary outcome. It was assessed using the question “Do you experience your personal situation as stressful at the moment?” with a five-point response scale (1 = “Does not apply at all” to 5 = “Applies completely”).

#### 2.3.2. Independent Variables

The spectrum of investigated independent variables can be grouped into eight topics.

(1) Education environment: Related questions included general queries about the field of study, the type of study and the current semester in which the participants were enrolled. Additional questions covered the confidence in the university’s handling of the pandemic and the likelihood of a delay in studies and its complications.
(2) Sociodemographic data: Participants were asked about their age, gender, relationship status and migration background. Questions regarding care for children or relatives were also included.
(3) Financial issues: All participants were asked to assess their economic situation and employment. Those who stated having a job were asked to indicate their management of work-study balance, average number of hours worked and dependence to cover living expenses.
(4) COVID-19 exposure: COVID-19 exposure was measured by a question on self-exposure and a question on cases and courses in the participants’ personal environment. The perceived likelihood for and severity of an infection were asked following the COVID-19-specific survey tool published by the WHO [21].
(5) General health: General health was assessed using a question on general health perception from the Short Form-36 Health Survey (SF-36) [22] and the single-item-scale for general life satisfaction (L-1) with a modified seven-point Likert scale [23]. Furthermore, changes in alcohol consumption, tobacco use and physical activity were collected.
(6) Mental health: To assess the tendencies to cope with stress, the Brief Resilience Coping Scale (BRCS) consisting of four items was used [24]. To evaluate social support, the Social Support Scale with four items from Satows’ Stress and Coping Inventory (SCI) was adduced with a modified five-point Likert scale [25]. The effect related to COVID-19 (worries and fears) was assessed using an item of the WHO survey tool [21]. The statement options were adapted to the context of the population of students.
(7) Daily life: Information on how the COVID-19 pandemic has affected students’ lives and how they perceive the changes were collected using questions of the German COVID-19 Snapshot Monitoring (COSMO) and items from a Swiss study performed on university students [26,27].
(8) Learning experiences: To assess experiences with online learning, students were asked about their technical equipment and knowledge required for successful participation in online classes. Furthermore, participants were asked to indicate their position on statements about comparisons between regular study periods and online learning. Study engagement was assessed using the Utrecht Work Engagement Scale for Students (UWES-9S) in which participants rate themselves on nine statements on a seven-point Likert scale [28].

### 2.4. Data Collection

The online survey was conducted from 29 June to 26 July 2020 using the SoSci Survey tool. The online questionnaire was offered in German and English and contained open and closed questions. A link to the questionnaire was sent to ~6600 students by university counsellors via email to recruit participants. Reminders were sent after the first week and in mid-July 2020.

### 2.5. Data Preparation

To impute missing data, the median and the mode for all nominal scaled variables were calculated over all cases of the respective variables. For standardized measures with only one item missing, the value was imputed using the median of the other items from the same case. Each variable of interest had less than 2% missing information. The calculations were performed using SAS^®^ University Edition (SAS Institute Inc., Cary, NC, USA).

The originally five-point scaled outcome was transformed into a dichotomous variable, with the two higher response options combined into “high burden” and the three lower response options summarized into “low burden”. The dichotomization of the variable was chosen to avoid a large number of beta estimators and thus improve the readability and applicability of the results.

As this paper focuses on factors that may be associated with high burden perception, the medium response category was chosen to be assigned to the low burden category in order to only allocate participants who stated to feel highly stressed during the COVID-19 pandemic to the outcome value of interest. For reasons of comparability, a supplementary analysis was performed using an alternative outcome in which the medium response option was assigned to “high burden”.

### 2.6. Data Analysis

Participants who completed at least 75% of the variables of interest were included in the analysis. Descriptive statistics were carried out to explore the distribution of variables. A simple percentage distribution was used to assess basic characteristics of the student population. The participants’ gender and age were reflected the distribution of these characteristics in the total student population enrolled in health sciences at LMU and KSH. Therefore, it was decided not to adjust for any sociodemographic factors.

Due to the binary scaling of the outcome, logistic regression was chosen for the analysis. Bivariate logistic regression was performed to select variables that significantly (with α ≤ 0.05) impacted the outcome. Multivariable logistic regression with stepwise selection was then performed to identify relevant variables for the final model.

To test the stability of the model, two different variable selection strategies were run. In the first version, all variables selected from the dataset were used to build multivariable regression models based on the eight predefined topics (education environment, sociodemographic data, financial issues, COVID-19 exposure, general health, mental health, daily life and learning experiences). Stepwise selection was performed for all eight models and the selected variables were taken to form a combined model. Stepwise selection was performed again on the resulting combined model, and the remaining variables were put into the final model. The second version used the same procedure, but only variables that were significant in the bivariate analyses were used for the selection process by category. Both selection versions resulted in the same set of variables that significantly impact the outcome.

The same strategy for creating the final regression model was performed using the alternative outcome. Details are available in Table S2 of the Supplementary Material. All analyses were performed using SAS^®^ University Edition (SAS Institute Inc., Cary, NC, USA).

## 3. Results

### 3.1. Participant Data

In total, 751 students started the questionnaire. Of those, 128 (17%) students quit with over 25% missing values for the variables of interest and were therefore excluded from the analyses. In total, 623 students (83%) answered at least 75% of the questions and were included in further analyses (response rate: 9%).

As shown in Table 1, study participants were predominantly female (*n* = 514; 83%) and between 18 and 24 years old (*n* = 308; 49%). Approximately two thirds of the participants were studying at KSH (*n* = 434; 70%) and one third at LMU (*n* = 189; 30%). In total, 37% of the study population was enrolled in the first year of their studies (*n* = 228), and most participants were studying full time (*n* = 529; 85%). Nearly half of the participating students (*n* = 275; 44%) reported feeling high levels of stress during the COVID-19 pandemic.

### 3.2. Main Results

The variable selection of 72 independent variables in total led to the identification of nine variables from different topics that demonstrating having a significant impact on psychological stress in the study population (see Table 2). The adjusted R^2^ for the regression was 0.6568. The supplementary analysis using an alternative outcome with a shifted topics constellation of the response options showed a similar selection of variables (see Table 3).

Regarding the highly significant variables from the final model, participants who were partially satisfied with their lives overall (*n* = 140; 22%), indecisive about their overall life satisfaction (*n* = 76; 12%), partially dissatisfied (*n* = 85; 14%) or dissatisfied (*n* = 51; 8%) showed a significantly increased chance of experiencing psychological stress compared to students who reported feeling completely satisfied (*n* = 61; 10%) (see Table 4).

In total, 375 of the students (72%) described their general health status before the COVID-19 pandemic as “excellent” or “very good”. At the time of the survey that number decreased by 14% to 291 students. Those who felt that their health situation had worsened during the pandemic (*n* = 212; 34%) were significantly more likely to perceive higher psychological stress (see Table 4) when compared to the students who reported no change in their current health situation in comparison to before the COVID-19 pandemic (*n* = 334; 54%). 

At the time of the survey, 481 students (77%) felt that a second wave was at least “partly likely”, and 64% (*n* = 401) experienced this at least as “rather stressful”. Compared to those who felt very stressed by the thought of a second wave, students who assigned themselves to the other response categories (“not a burden at all” to “stressful”) were less likely to feel stressed overall (see Table 4). Due to the small proportion (*n* = 23; 4%) who reported not feeling burdened at all by the thought of a second wave, the reverse results were not applicable.

Two significant study-related stressors were identified. The stress level of studies was highly significant (see Table 2), and the majority of students reported an increased level of study-related stress compared to previous semesters (*n* = 366; 59%), while for 23% (*n* = 141) it remained the same. Compared to those who expressed significantly higher levels of stress from their studies, the chances for high psychological stress were found to be lower for students in all other response clusters. The observed odds ratios were significant for all groups except for those who felt significantly less stressed from their studies (see Table 4). The reverse results were not applicable due to the small proportion (*n* = 31; 5%) who reported feeling significantly less burdened.

### 3.3. Supplementary Analysis

In the supplementary analysis, 11 variables demonstrated having a significant impact on psychological stress in the study population (see Table 3), and the adjusted R^2^ was 0.6811. Six of the selected variables were identical to the model described above (see Table 3; variables marked with ^§^). For the other three variables of the former model, similar variables from the same fields (social support, worry and perception) significantly affected the alternative outcome. Additional variables selected within this model were lower coping (*p* = 0.0221) and adequate technical equipment to participate in online courses (*p* = 0.0010). Detailed information on the alternative outcome model, selected variables and calculated estimates can be found in Table S2 of the Supplementary Material.

## 4. Discussion

This study investigated a wide variety of potential factors potentially pejorating psychological stress in a population of students in health-related fields at two universities in Munich, Germany, after the first wave of the COVID-19 pandemic. Looking at the results, first, it is noteworthy that our investigation indicated that the COVID-19 pandemic is associated with high psychological stress levels among students in health-related fields. Over four out of ten respondents experienced self-reported psychological stress. This rate is consistent with preliminary data reported among the German general population during the COVID-19 pandemic [29]. Furthermore, it appears to be higher than estimates obtained among health science students before the outbreak [30]. In fact, the results of a 2017 German nationwide student health survey found that 11% of students in health sciences reported suffering from depression, 15% reported having generalized anxiety, 22% reported symptoms of burnout and 26% reported experiencing high levels of stress [30]. Our survey showed elevated levels of psychological stress in 44% of the participants, which is similar to the results of studies among higher education students from other countries during the COVID-19 pandemic [14,16]. The results suggest that students’ mental health, as represented by self-reported psychological stress, is significantly linked to matters in students’ personal lives as well as study-related concerns.

Regarding aspects in students’ personal lives, our results on *general health status* are in line with Lai et al. who found personal health status to be associated with higher perceived stress levels and more severe anxiety and depression symptoms during the COVID-19 pandemic in international university students. They also identified the health of friends and family as a COVID-19-related stressor [31], which was not surveyed by the questionnaire used in this study and thus could not be included in the analysis. However, our survey captured concerns about the health of friends and family which was not found to be associated with psychological stress. Coyle et al. identified physical activity as a coping strategy to positively influence students’ mental well-being during the COVID-19 pandemic [32], and other studies found that low levels of physical activity are a risk factor for distress, depression and anxiety in students [16,33]. However, the questionnaire used for this study only assessed changes in physical activity that had no significant impact on psychological stress.

Low *overall satisfaction with life* was associated with high psychological stress, and the percentage of students who reported not being satisfied with their lives is worrying. Only 65% of the study population reported feeling at least “partly satisfied” with their lives overall, which is in sharp contrast to the 2017 German survey on student health, in which 84% of health science students agreed with the statement [30]. This downward trend is important, as Aslan et al. also found satisfaction to be negatively correlated with perceived stress and the perceived impact of the COVID-19 pandemic [33].

The worsened trend of dissatisfaction might partly be affected by decreased social interactions due to physical distancing during the COVID-19 pandemic, which have been described as a risk factor for increased stress and anxiety in students [34]. Most students reported missing *contact with their fellow students*, which is in line with the findings of several studies that have identified social isolation as a risk factor for psychological stress [16,34]. Social support is another important environmental resource for individuals and closely related to mental health [35]. In line with previous studies [14,31,36,37,38], this analysis has shown high levels of stress in students to be significantly associated with a lack of *social support* when feeling stressed. In maintaining higher education students’ mental health, attention should thus be paid to social support structures and interactions. One way for universities to act would be to establish online mental health education courses related to the COVID-19 pandemic to improve their students’ psychological adaptability. This could also help students to develop skills to contain the fear of a worsening of the situation. The survey took place after the first wave of the COVID-19 pandemic, and the *fear of a second wave*, which started occurring in Germany during the winter [39], was a risk factor for increased stress. Therefore, it is important to address students experiencing higher levels of stress and ensure their psychological well-being. This seems to be especially important, as a Swiss study showed that the effects of the second wave had an even greater impact on peoples’ mental health status than the first wave [40].

It has been previously shown that financial vulnerability may exacerbate distress among students [41]. Fu et al. found low economic status to be associated with anxiety symptoms during the COVID-19 pandemic [14], and loss of income has also been identified as a risk factor by other studies [16,36]. However, this analysis showed that even the mere *worry of getting into financial difficulties due to loss of income* had a significant impact on psychological stress. Students are often marginally employed in gastronomy or retail: employment sectors which were severely affected by the COVID-19 pandemic. Therefore, students were especially at risk of losing their sources of income.

The study showed that participants who felt they could do nothing to positively affect the situation, indicating a high external locus of control, were more likely to experience high levels of psychological stress. Even before the pandemic a significant negative relationship between perceived stress and locus of control has been reported among higher education students [42]. It can be assumed that the effects have worsened, which is in line with Mudenda et al. who found that feelings of helplessness during the COVID-19 pandemic were associated with mental health in pharmacy students [43]. Consistent with this observation, Kuehner et al. found a high internal locus of control to be a conducive personality trait for better psychological well-being during the COVID-19 pandemic [44]

With respect to *study-related concerns*, the present study showed that an increased likelihood of a *prolonged study duration* was associated with a higher level of psychological stress. This is in line with similar results by Cao et al. who also found that next to the effects on daily life and economic effects, delays in academic activities were associated with anxiety symptoms [36]. A U.S. study on students’ mental health during the COVID-19 pandemic also indicated that increased concerns about academic performance were associated with stress and anxiety [34]. Additionally, a German study concerning medical students found a significant impact of academic context on higher perceived distress levels [16]. Lai et al. determined a similar association of academic performance and perceived stress. Additionally, they found that stress related to uncertainty about the academic program was also associated with higher perceived stress levels and more severe symptoms of mental disorders [31]. For study-related factors, we also found that compared to the usual study format, a lower *perceived stress level from the study load* was a protective factor for the development of stress. From the results, it can be concluded that universities should be advised to adjust learning plans to the current challenges of online learning and adapt their schedule to workload changes in order to reduce students’ psychological stress.

Overall, the results of this study also suggest that the impact of the COVID-19 pandemic on students’ mental health should also be considered more in policy discourse. The results of a longitudinal study from Germany showed that young people are particularly burdened by the current situation [45], and almost one in two 15- to 30-year-olds felt that their concerns were either not heard or not requested [46]. Thus, although the younger population is least likely to experience a severe or critical course when infected with the coronavirus [11], the long-term consequences of mental health deterioration could be severe, and actions must be taken to ensure students’ psychological well-being. 

Building on our findings, we recommend further research to examine the coping mechanisms students used during the COVID-19 pandemic when faced with the variety of pandemic-related challenges. In addition, it is also necessary to explore how students dealt with the negative consequences of social isolation, loneliness and financial hardship. For this purpose, we recommend using methods such as qualitative study designs to gain in-depth insight and understanding.

Some limitations should be considered when interpreting the obtained results. First, although the number of respondents is large, it represents 9% of the students contacted, and there is a possible response and desirability bias that may have altered the results. However, it has been shown that low response rates in epidemiological surveys only marginally affect prevalence and association measures [47]. Additionally, the invitation to participate was sent out via the university’s distribution list. Therefore, only students who had survey participation notifications enabled in their settings were reached, which may pose a potential source of self-selection bias. As we focused on students from health-related fields, the results are not generalizable to students in all disciplines. Furthermore, this study used a self-report questionnaire, and the data obtained reflect the subjective perception of the participants; generalized anxiety and depression using validated and standardized questionnaires were not assessed. The transformation of psychological stress must be taken into account. However, since the alternative model provided comparative results, the stability of the results can be assumed. In a follow-up study, the same participants should be surveyed to determine the persistence (or transience) of psychological stress. The results must also be considered in the context of a nonacute phase of the pandemic while lockdown measures were comparatively relaxed. The survey was conducted during the exam period at both universities. Due to the cross-sectional design of the study, it cannot be assured that the reported results are related to the COVID-19 pandemic or whether the distribution of psychological stress was preexisting or influenced also by the study period. However, a longitudinal study found that stress levels continued to be elevated at similar levels after the initial lockdown [48]. A follow-up study using the same questionnaire could help to validate and substantiate the results found. Further analyses could also examine differences among universities.

Despite these limitations, the present study has important strengths. First, a sample with over 600 students in health-related fields from two different universities was investigated. Second, a variety of demographic, financial, health-related, motivational and teaching-related factors were analyzed to strengthen the perspectives of students regarding their mental burden during the COVID-19 pandemic.

## 5. Conclusions

Protecting the mental health of students in general is a public health issue that appears even more critical in the context of a pandemic. Therefore, attention should be paid to the psychological well-being of young adults, and the negative effects of lockdown measures should be considered in policy making. This is especially important as the study was conducted after the first lockdown in Germany. Long-term quarantine due to the COVID-19 pandemic may lead to further deterioration in the psychological well-being of students. Further efforts should investigate the relationship of reported high level of psychological stress and the COVID-19 pandemic as well as the coping strategies applied and their effect on students’ mental health.

Our findings suggest that special attention needs to be paid to students with low life satisfaction levels and low social and financial support structures. Therefore, universities should encourage faculty members to maintain contact with students, pay attention to their (mental) health, enable them to maintain social ties and support them in their studies by providing flexibility in structure and adjusting the workload to meet the current challenges of online learning. Findings obtained from this study combined with those from similar studies are crucial to implement timely and appropriate interventions for students at risk to reduce the psychological harm caused by the COVID-19 pandemic. Interventions could include digital forms of study groups, peer group sessions, regular online consultation hours, mentoring and psychological counselling [49]. Further investigations are necessary to evaluate interventions that respond most appropriately to student’s needs.

## Figures and Tables

**Table 1:** Characteristics of the study population.

Variable	Frequency (*n*)	Percentage
Total	623	100
Gender		
Female	514	82.50
Male	105	16.86
Divers	4	0.64
Age		
18–24 years	308	49.44
5–29 years	156	25.04
30–34 years	82	13.16
>34 years	77	12.36
University		
LMU Munich	189	30.34
KSH	434	69.66
Enrolled year of studies		
1	228	36.60
2	113	18.14
3	175	28.09
4	74	11.88
5	28	4.49
6	5	0.80
Study form		
Full-time	529	84.91
Part-time	94	15.09
Psychological stress		
Low	348	55.86
High	275	44.14

**Table 2:** Final model.

Variable	DF ^1^	*p*-Value
Life satisfaction	6	<0.0001 **
General health status	2	<0.0001 **
Social support (stress/pressure)	4	0.0301 *
Worries about loss of income	7	0.0134 *
Stressful thoughts on a second wave	6	<0.0001 **
Perception: rating current situation as uninfluenceable	6	0.0262 *
Reduced social contact	5	0.0115 *
Delay of studies	4	0.0178 *
Stress level of studies	4	0.0003 **

^1^ DF = Degrees of freedom. * = significant at α ≤ 0.05. ** = highly significant at α ≤ 0.001.

**Table 3:** Alternative outcome model.

Variable	DF ^1^	*p*-Value
*Life Satisfaction* ^§^	6	<0.0001 **
*General health status* ^§^	2	0.0003 **
Coping	2	0.0221 *
Social support (bad situation)	4	0.0200 *
Worries about employment	7	0.0002 **
*Stressful thoughts on a second wave* ^§^	6	0.0008 **
Perception: boredom	6	0.0086 *
*Reduced social contact* ^§^	5	0.0319 *
Adequate technical equipment	6	0.0010 *
*Delay of studies* ^§^	4	0.0067 *
*Stress level of studies* ^§^	4	<0.0001 **

^1^ DF = Degrees of freedom. * = significant at α ≤ 0.05. ** = highly significant at α ≤ 0.001. ^§^ Variables identical to the main model (see Table 2).

**Table 4:** Final regression model.

Variable	Class Value	Reference Class	Estimate	OR	Standard Error	*p*-Value	Lower CI 95%	Upper CI 95%
Intercept			−3.4979		1.1119	0.0017 *		
Life satisfaction How satisfied are you currently—all in all—with your life?	Satisfied	Completely satisfied	1.1662	3.210	0.6356	0.0665	0.924	11.155
	Partly satisfied		1.6559	5.238	0.6489	0.0107 *	1.468	18.686
	Partly dissatisfied/partly satisfied		3.1536	23.419	0.7167	<0.0001 **	5.748	95.411
	Partly dissatisfied		3.6386	38.037	0.7295	<0.0001 **	9.105	158.902
	Dissatisfied		3.0875	21.922	0.7875	<0.0001 **	4.683	102.613
	Completely dissatisfied		2.6403	14.017	1.4988	0.0781	0.743	264.527
General health status Current state of health in comparison to before the COVID-19 pandemic.	Improved health situation	No change	−0.8256	0.438	0.4603	0.0728	0.178	1.079
	Worsened health situation		1.1050	3.019	0.2849	0.0001 **	1.727	5.277
Social support (stress/pressure) Under stress and pressure, I find support from my partner or a good friend.	Does not apply at all	Applies completely	−0.8031	0.448	1.0135	0.4281	0.061	3.265
	Rather not true		1.6830	5.382	0.6748	0.0126 *	1.434	20.199
	Partly applies		0.6485	1.913	0.4744	0.1716	0.755	4.846
	Applies		0.5749	1.777	0.2852	0.0438 *	1.016	3.108
Worries about loss of income Given the current COVID-19 situation, how worried are you that you get into financial difficulties due to loss of income?	Few concerns	Very few concerns	0.4768	1.611	0.3868	0.2176	0.755	3.438
	Rather few concerns		0.1648	1.179	0.4233	0.6970	0.514	2.703
	Partly		−0.5080	0.602	0.4662	0.2759	0.241	1.500
	Rather concerns		1.2615	3.531	0.4787	0.0084 *	1.382	9.022
	Concerns		0.8447	2.327	0.5104	0.0979	0.856	6.329
	Greatest concerns		−0.0980	0.907	0.6573	0.8815	0.250	3.288
	Not relevant		1.3293	3.778	0.5100	0.0092 *	1.390	10.268
Stressful thoughts on a second wave How stressful is the thought of a second wave for you?	Not a burden at all	Very stressful	−1.6920	0.184	0.7121	0.0175 *	0.046	0.744
	Not a burden		−3.2120	0.040	0.6594	<0.0001 **	0.011	0.147
	Rather not a burden		−1.5877	0.204	0.5487	0.0038 *	0.070	0.599
	Partly not a burden/partly stressful		−0.8792	0.415	0.4598	0.0559	0.169	1.022
	Rather stressful		−1.3874	0.250	0.4043	0.0006 **	0.113	0.552
	Stressful		−0.8271	0.437	0.3925	0.0351 *	0.203	0.944
Perception: rating the current situation as uninfluenceable I myself can do nothing to positively influence the situation.	Not true	Does not apply at all	−0.0399	0.961	0.3433	0.9074	0.490	1.883
	Rather not true		0.2866	1.332	0.4014	0.4752	0.606	2.925
	Partly applies		1.1238	3.077	0.4264	0.0084 *	1.334	7.096
	Rather applies		0.9443	2.571	0.5176	0.0681	0.932	7.090
	Applies		1.4341	4.196	0.7079	0.0428 *	1.048	16.804
	Applies completely		0.8331	2.300	0.7987	0.2969	0.481	11.008
Reduced social contact I miss social contact with my fellow students.	Do not agree	Do not agree at all	0.4389	1.551	1.0584	0.6784	0.195	12.347
	Partly agree		−0.4838	0.616	0.9233	0.6003	0.101	3.765
	Agree		1.2976	3.660	0.8341	0.1198	0.714	18.770
	Completely agree		1.3566	3.883	0.7838	0.0835	0.836	18.045
Delay of studies How likely do you think it is that your studies/career will be delayed by the COVID-19 pandemic?	Not likely	Not likely at all	0.8177	2.265	0.3907	0.0363 *	1.053	4.872
	Partly not likely/partly likely		1.2019	3.326	0.3994	0.0026 *	1.520	7.278
	Likely		0.8261	2.284	0.4117	0.0448 *	1.019	5.119
	Very likely		1.3835	3.989	0.4743	0.0035 *	1.574	10.106
Stress level of studies How high do you estimate your current level of stress from your studies compared to your usual format?	Significantly less	Significantly higher	−0.00609	0.994	0.7761	0.9937	0.217	4.550
	Less		−1.6972	0.183	0.4721	0.0003 **	0.073	0.462
	Partly less/partly higher		−1.4546	0.233	0.3797	0.0001 **	0.111	0.491
	Higher		−0.8370	0.433	0.3309	0.0114 *	0.226	0.828

* = significant at α ≤ 0.05. ** = highly significant at α ≤ 0.001.

## Data Availability

The datasets generated and analyzed during the current study are available from the corresponding authors on reasonable request.

## Supplementary Materials

The following materials are available online at https://www.mdpi.com/article/10.3390/ijerph18126611/s1, Table S1: Overview of independent variables. Table S2: Alternative Outcome Regression Model.
