# Supplementary material for: Psychological Stress among Students in Health-Related Fields during the COVID-19 Pandemic: Results of a Cross-Sectional Study at Selected Munich Universities
Source: Int J Environ Res Public Health. 2021 Jun 19;18(12):6611. doi: 10.3390/ijerph18126611 (PMC8296436; doi:10.3390/ijerph18126611)
Supplement: Supplementary file 1 [file ijerph-18-06611-s001.zip › ijerph-1242489-supplementary.pdf]

Table S1. Overview of independent variables

Topic	Variable / *Questionnaire*	Question	Response Options	Reference
Education environment	Affiliated university	Which college / university do you study at?	Binary response option „Ludwig-Maximilians-Universität Munich (LMU)“ / „Katholische Stiftungshochschule Munich (KSH)“	1
	Subject of study	Which subject are you studying?	[01] Medicine<br>[02] Dentistry<br>[03] Public Health (M.Sc.)<br>[04] Epidemiology (M.Sc.)<br>[05] Social work (B.A.)<br>[06] Social work part-time (B.A.)<br>[07] Children education (B.A.)<br>[08] Children education part-time (B.A.)<br>[09] Healthcare-Management (B.A.)<br>[10] Nursing management (B.A.)<br>[11] Nurse education (B.A.)<br>[12] Nursing dual (B.Sc.)<br>[13] Midwifery education (B.Sc.)<br>[14] Nursing (B.Sc.)<br>[15] Applied social and educational sciences (M.A.)<br>[16] Management of social and health companies (M.A.)<br>[17] Applied Health Services Research (M.Sc.)<br>[18] Education and training management in the health system (M.A.)<br>[19] Nursing Science - Innovative Care Concepts (M.Sc.)	1
	Enrolled semester	In which semester of your current studies are you enrolled?	[number]	1
	Form of study	Which form of study applies to you?	Binary response option “Full-time” / “Part-time”	1
	Trust in university handling current situation	How do you think your college / university deals with the current situation?	5 level Likert scale “Very good” – “Very bad”	1
	Informed about university’s decisions	I am well informed about the consequences of the decisions of LMU Munich/ KSH Munich regarding my semester examinations.	5 level Likert scale with additional option for “not relevant” “Do not agree at all” – “Completely agree”	2
Sociodemographic data	Age	How old are you?	[01] <18 years<br>[02] 18-24 years<br>[03] 25-29 years<br>[04] 30-34 years<br>[05] >34 years	1

Topic	Variable / *Questionnaire*	Question	Response Options	Reference
	Gender	I identify my gender as:	[01] Female<br>[02] Male<br>[03] Divers	[1]
	Relationship	Are you in a relationship or partnership?	Binary response option "yes" / "no"	[3]
	Children	Do you live with your own children or children of your partner in the same household?	Binary response option "yes" / "no"	[1]
	Single Parent	Are you a single parent?	Binary response option "yes" / "no"	[3]
	Care of relatives	Do you currently care for relatives who are in need of care due to age or illness?	Binary response option "yes" / "no"	[3]
	Migration background	Do you have a migration background?<br>Note: In Germany, a person has a migration background if he or she or at least one parent was not born with German citizenship.	Binary response option "yes" / "no"	[1]
Financial issues	Employment	Do you have one or more job(s) during the lecture period with which you earn money?	[01] No<br>[02] Yes, one job<br>[03] Yes, two different jobs<br>[04] Yes, three or more different jobs	[4] modified
	Number of jobs	Number of jobs in different sectors	[number]	[4] modified
	Type of employment	(multiple answers possible)	[01] Job in the health sector requiring a vocational training qualification or university degree<br>[02] Job in social services requiring a vocational training qualification or university degree<br>[03] Job requiering a vocational training qualification or university degree<br>[04] Job as a student assistant / research assistant<br>[05] Jobbing (e.g. in a factory, an office, a pub, babysitting)	[4] modified
	Dependency for living expenses	Are you dependent on paid employment to cover your study and living costs (tuition fees, food, rent, etc.)?	Binary response option "yes" / "no"	[2]
	Working hours before pandemic	To what extent did you work during the lecture period before the Corona pandemic?	[in hours]<br>[number]	[5] modified
	Working hours under current conditions	To what extent can you pursue your gainful employment under the current pandemic conditions?	[in hours]<br>[number]	[5] modified
	Agreement work and studies	How well do you currently manage to reconcile studying and working?	5 level Likert scale "Very good" – "Very bad"	[6] modified

Topic	Variable / *Questionnaire*	Question	Response Options	Reference
	Overall economic situation	How do you assess your current economic situation compared to the time before the Corona pandemic?	5 level Likert scale “Very good” – “Very poor”	[1]
COVID-19 exposure	Infection	Have or had you been infected with the novel coronavirus?	[01] Yes, the infection is confirmed.<br>[02] Yes, but the infection hasn't been confirmed yet.<br>[03] Yes, but the infection has already passed.<br>[04] No	[7]
	Infection in social environment	Fallback option (negative) or number of selected options	[number]<br>[-01] I don’t now	[7]
	Type of infection in social environment	(multiple answers possible)	[01] There are untested suspected cases<br>[02] There are confirmed cases of infected people<br>[03] There are recovering people<br>[04] There are deceased people<br>[05] There are no cases	[7]
	Probability for an infection	How high do you estimate your risk of being infected with the novel coronavirus?	7 level Likert scale “Extremely unlikely” – “Extremely likely”	[3]
	Severity of an infection	How do you assess a possible infection with the novel coronavirus for yourself?	7 level Likert scale “Completely harmless” – “Extremely dangerous”	[3]
	Second wave probability	How likely is the scenario of a second wave of the corona virus pandemic in the future in your opinion?	7level Likert scale “Extremely unlikely” – “Extremely likely”	[3] modified
General health	Health status under current conditions	How would you describe your current state of health in general?	5 level Likert scale “Excellent” – “Bad”	[8]
	Health status before pandemic	How would you describe your state of health before the corona pandemic in general?	5 level Likert scale “Excellent” – “Bad”	[8]
	Alcohol consumption	Has your alcohol consumption behaviour changed since the beginning of the Corona pandemic?	[01] Yes, I consume more.<br>[02] Yes, I consume less.<br>[03] Yes, I started drinking alcohol during the Corona pandemic.<br>[04] No, I consume as much as usual.<br>[05] No, I don't drink alcohol in general.	[1]
	Smoking behaviour	Has your smoking behaviour changed since the beginning of the Corona pandemic?	[01] Yes, I consume more.<br>[02] Yes, I consume less.<br>[03] Yes, I started smoking in the Corona pandemic.<br>[04] No, I consume as much as usual.<br>[05] No, I don't smoke in general.	[1]

Topic	Variable / *Questionnaire*	Question	Response Options	Reference
	Physical activity	Has your intensive physical activity changed since the beginning of the Corona pandemic? (e.g. fast cycling, jogging, swimming or other physical activities)	[01] Yes, I'm more active.<br>[02] Yes, I'm less active.<br>[03] Yes, I started using during the Corona pandemic.<br>[04] No, I'm exercising as much as ever.<br>[05] No, I'm not getting any exercise at all.	[1]
Mental health	Life satisfaction	How satisfied are you currently - all in all - with your life?	7 level Likert scale "Completely satisfied" – "Completely dissatisfied"	[9] modified
	Brief Resilience Scale		5 level Likert scale "Does not apply at all" – "Applies completely"	[10]
	Stress- und Coping-Inventar		5 level Likert scale "Does not apply at all" – "Applies completely"	[11]
	Worries about losing beloved person	Given the current Corona situation, how worried are you that you lose someone you love?	7 level Likert scale with additional option for "not relevant" "Very few concerns" – "Greatest concerns"	[7]
	Worries about friend's health	Given the current Corona situation, how worried are you that a friend is / will be affected by health problems?	7 level Likert scale with additional option for "not relevant" "Very few concerns" – "Greatest concerns"	[7]
	Worries about family's health	Given the current Corona situation, how worried are you that a family member is / will be affected by health problems?	7 level Likert scale with additional option for "not relevant" "Very few concerns" – "Greatest concerns"	[7]
	Worries about a friend being economically affected	Given the current Corona situation, how worried are you that a friend is / will be economically affected?	7 level Likert scale with additional option for "not relevant" "Very few concerns" – "Greatest concerns"	[7]
	Worries about a family member being economically affected	Given the current Corona situation, how worried are you that a family member is / will be economically affected?	7 level Likert scale with additional option for "not relevant" "Very few concerns" – "Greatest concerns"	[7]
	Worries about the health system	Given the current Corona situation, how worried are you that the health system is overburdened?	7 level Likert scale with additional option for "not relevant" "Very few concerns" – "Greatest concerns"	[7]
	Worries about studies	Given the current Corona situation, how worried are you that you are / will be influenced in the course of your studies?	7 level Likert scale with additional option for "not relevant" "Very few concerns" – "Greatest concerns"	[7] modified
	Worries about employment	Given the current Corona situation, how worried are you that you lose your job? (if applicable)	7 level Likert scale with additional option for "not relevant" "Very few concerns" – "Greatest concerns"	[7]

Topic	Variable / *Questionnaire*	Question	Response Options	Reference
	Worries about loss of income	Given the current Corona situation, how worried are you that you get into financial difficulties due to loss of income?	7 level Likert scale with additional option for "not relevant" "Very few concerns" – "Greatest concerns"	7
	Thoughts on a second wave	How stressful is the thought of a second wave for you?	7 level Likert scale "Not a burden at all" – "Very stressful"	1
Daily life	Contact through digital devices	I call or talk with family, friends and acquaintances via digital devices.	7 level Likert scale "Does not apply at all" –"Applies completely"	3
	Receiving support	I receive support offers from family, friends or neighbours.	7 level Likert scale "Does not apply at all" –"Applies completely"	3
	Offering support	I offer help to others, e.g. neighbourhood help with shopping.	7 level Likert scale "Does not apply at all" –"Applies completely"	3
	Daily routine	I have a plan for my everyday life in terms of sleep, work or physical activities.	7 level Likert scale "Does not apply at all" –"Applies completely"	3
	Perception: boredom	I'm bored.	7 level Likert scale "Does not apply at all" –"Applies completely"	3
	Perception: rating current situation as uninfluenceable	I myself can do nothing to positively influence the situation.	7 level Likert scale "Does not apply at all" –"Applies completely"	3
	Social contact	I miss the social contacts with my fellow students.	5 level Likert scale with additional option for "not relevant" "Do not agree at all" – "Completely agree"	2
	Contact to lecturers	The lecturers are there as contact persons for the students.	5 level Likert scale with additional option for "not relevant" "Do not agree at all" – "Completely agree"	2
	Planned internships	I can do my internships as planned (during my studies or summer holidays).	5 level Likert scale with additional option for "not relevant" "Do not agree at all" – "Completely agree"	2
	Confidence mastering study requirements	I have the feeling that I can master my study requirements with confidence.	5 level Likert scale with additional option for "not relevant" "Do not agree at all" – "Completely agree"	2
Learning experiences	Adequate technical equipment	My technical equipment is sufficient to participate in digital courses as planned.	7 level Likert scale "Does not apply at all" – "Applies completely"	12
	Adequate technical knowledge	My technical knowledge is sufficient to participate in digital courses as planned.	7 level Likert scale "Does not apply at all" – "Applies completely"	12
	Time flexibility	The digital teaching offers me greater flexibility in terms of time.	5 level Likert scale with additional option for "not relevant" "Do not agree at all" – "Completely agree"	13
	Spatial flexibility	The digital teaching offers me greater spatial flexibility.	5 level Likert scale with additional option for "not relevant" "Do not agree at all" – "Completely agree"	13

Topic	Variable / *Questionnaire*	Question	Response Options	Reference
	Response to individual needs	Digital teaching responds better to my individual needs.	5 level Likert scale with additional option for "not relevant" "Do not agree at all" – "Completely agree"	13
	Communication with teachers	Communication with teachers is better in digital teaching.	5 level Likert scale with additional option for "not relevant" "Do not agree at all" – "Completely agree"	13
	Opportunities for interaction and participation	Digital teaching offers better opportunities for interaction and participation.	5 level Likert scale with additional option for "not relevant" "Do not agree at all" – "Completely agree"	13
	Worries about degree	I'm worried about my university degree.	5 level Likert scale with additional option for "not relevant" "Do not agree at all" – "Completely agree"	2
	Stress level of studies	How high do you estimate your current level of stress from your studies compared to your usual format?	5 level Likert scale with additional option for "not relevant" "Significantly less" – "Significantly higher"	1
	Delay of studies	How likely do you think it is that your studies / career will be delayed by the Corona pandemic?	5 level Likert scale "Not likely at all" – "Very likely"	6 modified
	Effect of delay of studies	How bad would such a delay be for you?	5 level Likert scale "Not bad at all" – "Very bad"	1
	Utrecht Work Engagement Scale		7 level Likert scale "Never" – "Always"	14

[1] = According to own development

[2] = Dratva, J. Studie zur Gesundheit von Studierenden in Zeiten der Corona-Pandemie. 2020. Available online: https://www.zhaw.ch/de/gesundheit/forschung/gesundheitswissenschaften/projekte/studierendengesundheit/ (accessed on 07 May 2020)

[3] = Betsch, C.; Korn, L.; Felgendreff, L.; Eitze, S.; Schmid, P.; Sprengholz, P.; Wieler, L.; Schmich, P.; Stollorz, V.; Ramharter, M.; Bosnjak, M.; Omer, S. B.; Thaiss, H.; De Bock, F.; Von Rüden, U.; Lämmlin, G.; Ahrens, P.-A. German COVID-19 Snapshot Monitoring (COSMO) - Welle 7. *PsychArchives*, **2020**. doi:10.23668/PSYCHARCHIVES.2875.

[4] = Middendorff, E. Deutsches Zentrum für Hochschul- und Wissenschaftsforschung (DZHW). 21. Sozialerhebung des Deutschen Studentenwerks durchgeführt vom Deutschen Zentrum für Hochschul- und Wissenschaftsforschung. 2016. Available online: http://www.sozialerhebung.de/download/21/Soz21_fragebogen_bi_deutsch.pdf (accessed on 07 May 2020)

[5] = Buß, I. Fragebogen zur Erhebung der strukturellen Studierbarkeit und studienstrukturellen Präferenzen mit Fokus auf berufstätige Studierende und Studierende mit Kindern. 2015. Available online: https://www.hwg-lu.de/fileadmin/user_upload/service/studium-und-lehre/Offenes_Studienmodell/Fragebogen_Studienstrukturen_HSLU_final.pdf (accessed on 07 May 2020)

[6] = Gartmeier, M. COVID19 – Wie geht es Ihnen?. 2020. Available online: https://www.meditum.med.tum.de/de/content/covid19-–-wie-geht-es-ihnen-bitte-um-studienteilnahme (accessed 26 May 2020)

[7] = World Health Organization. Regional Office for Europe. Survey Tool and Guidance. Rapid simple, flexible behavioural insights on COVID-19. 2020. Available online: https://www.euro.who.int/__data/assets/pdf_file/0007/436705/COVID-19-survey-tool-and-guidance.pdf?ua=1 (accessed on 06 May 2020)

[8] = Bullinger, M.; Kirchberger, I. SF-36, Fragebogen zum Gesundheitszustand. Hogrefe: Göttingen [u.a.], Germany, 1998.

[9] = Beierlein, C.; Kovaleva, A.; László, Z.; Kemper, C. J.; Rammstedt, B. Eine Single-Item-Skala zur Erfassung der Allgemeinen Lebenszufriedenheit: Die Kurzskala Lebenszufriedenheit-1 (L-1). *GESIS–Working Papers*, **2014**, 33.

[10] = Smith, B. W.; Dalen, J.; Wiggins, K.; Tooley, E.; Christopher, P.; Bernard, J. The Brief Resilience Scale: Assessing the ability to bounce back. *International Journal of Behavioral Medicine*, **2008**, 15(3), 194-200. doi:10.1080/10705500802222972.

[11] = Satow, L. Stress- und Coping-Inventar (SCI). Test- und Skalendokumentation. 2012. Available online: http://www.drsatow.de/tests/stress-und-coping-inventar/ (accessed on 07 May 2020)

[12] = Universität Greifswald. Studierendenbefragung zur digitalen Lehre. 2020. Available online: https://www.uni-greifswald.de/storages/uni-greifswald/2_Studium/2.1_Studienangebot/2.1.4_Qualitaet_in_Studium_und_Lehre/Stabsstelle_Integrierte_Qualitaetssicherung/Musterfragebogen_digitale_Lehre.pdf (accessed on 06 May 2020)

[13] = Barnes & Noble Education Inc. Barnes & Noble Education Survey Reveals College Student Preparedness Split: Technically Ready for Online Learning, But Emotionally Unsure. 2020. Available online: https://www.businesswire.com/news/home/20200408005156/en/ (accessed on 06 May 2020)

[14] = Schaufeli, W., & Bakker, A. UWES Utrecht Work Engagement Scale. Preliminary Manual. 2004. Available online: https://www.wilmarschaufeli.nl/publications/Schaufeli/Test%20Manuals/Test_manual_UWES_English.pdf (accessed on 07 May 2020)

Table S2. Alternative Outcome Regression Model

Variable	Class value	Reference class	Estimate	OR	Standard error	P-value	Lower CI 95%	Upper CI 95%
Intercept			-3.8663		1.1802	0.0011		
Life satisfaction<br>How satisfied are you currently - all in all - with your life?	Satisfied	Completely satisfied	-0.6260	0.535	0.4415	0.1562	0.225	1.270
	Partly satisfied		0.2541	1.289	0.4727	0.5909	0.510	3.256
	Partly dissatisfied / partly satisfied		1.7888	5.982	0.6222	0.0040*	1.767	20.253
	Partly dissatisfied		2.7263	15.276	0.9083	0.0027*	2.576	90.600
	Dissatisfied		0.3170	1.373	0.7588	0.6761	0.310	6.076
	Completely dissatisfied		1.0889	2.971	1.7609	0.5363	0.094	93.706
General health<br>Current state of health in comparison to before the Corona pandemic.	Improved health situation	No change	-0.2526	0.777	0.4082	0.5359	0.349	1.729
	Worsened health situation		1.4030	4.067	0.3652	0.0001**	1.988	8.321
Coping<br>Brief resilient coping scale.	Low resilient coper	High resilient coper	0.7757	2.172	0.4124	0.0600	0.968	4.874
	Medium resilient coper		0.7930	2.210	0.3048	0.0093*	1.216	4.016
Social support (bad situation)<br>No matter how bad it gets, I have good friends I can always count on.	Does not apply at all	Applies completely	18.1056	>999.999	1185.0	0.9878	<0.001	>999.999
	Rather not true		1.0433	2.839	0.8310	0.2093	0.557	14.469
	Partly applies		0.8360	2.307	0.4822	0.0829	0.897	5.936
	Applies		1.0431	2.838	0.3346	0.0018*	1.473	5.468
Worries about employment<br>Given the current Corona situation, how worried are you that you lose your job?	Few concerns	Very few concerns	1.0148	2.759	0.4289	0.0180*	1.190	6.394
	Rather few concerns		-1.0084	0.365	0.5917	0.0883	0.114	1.163
	Partly		-0.3361	0.715	0.6447	0.6022	0.202	2.528
	Rather concerns		1.6175	5.040	0.6384	0.0113*	1.442	17.614
	Concerns		1.7855	5.962	1.0173	0.0792	0.812	43.784
	Greatest concerns		1.4345	4.198	1.0865	0.1867	0.499	35.303
	Not relevant		1.4936	4.453	0.4241	0.0004**	1.939	10.226

Variable	Class value	Reference class	Estimate	OR	Standard error	P-value	Lower CI 95%	Upper CI 95%
Thoughts on a second wave<br>How stressful is the thought of a second wave for you?	Not a burden at all	Very stressful	-2.0491	0.129	0.7981	0.0102*	0.027	0.616
	Not a burden		-2.6795	0.069	0.6269	<.0001**	0.020	0.234
	Rather not a burden		-1.9352	0.144	0.5830	0.0009**	0.046	0.453
	Partly not a burden / partly stressful		-1.2082	0.299	0.5380	0.0247*	0.104	0.858
	Rather stressful		-1.3573	0.257	0.5083	0.0076*	0.095	0.697
	Stressful		-1.0406	0.353	0.5240	0.0470*	0.126	0.987
Perception: boredom<br>I'm bored.	Not true	Does not apply at all	0.6081	1.837	0.3893	0.1183	0.856	3.940
	Rather not true		0.9361	2.550	0.4845	0.0533	0.987	6.590
	Partly applies		1.0275	2.794	0.5244	0.0501	1.000	7.810
	Rather applies		1.5045	4.502	0.5260	0.0042*	1.606	12.622
	Applies		0.9384	2.556	0.8282	0.2572	0.504	12.957
	Applies completely		2.2450	9.440	0.8283	0.0067*	1.862	47.863
Reduced social contact<br>I miss the social contacts with my fellow students.	Do not agree	Do not agree at all	1.1519	3.164	1.0439	0.2698	0.409	24.480
	Partly agree		0.4692	1.599	0.9134	0.6075	0.267	9.578
	Agree		1.3736	3.950	0.8604	0.1104	0.731	21.328
	Completely agree		1.8579	6.410	0.8258	0.0245*	1.270	32.346
	Not relevant		2.6612	14.313	2.8561	0.3515	0.053	>999.999
Adequate technical equipment<br>My technical equipment is sufficient to participate in digital courses as planned.	Does not apply at all	Applies completely	12.1635	>999.999	990.2	0.9902	<0.001	>999.999
	Not true		3.9050	49.652	1.6282	0.0165*	2.042	>999.999
	Rather not true		1.7442	5.721	0.8505	0.0403*	1.080	30.298
	Partly applies		-0.7555	0.470	0.9118	0.4074	0.079	2.806
	Rather applies		-1.7058	0.182	0.4946	0.0006**	0.069	0.479
	Applies		-0.0932	0.911	0.3423	0.7854	0.466	1.782

Variable	Class value	Reference class	Estimate	OR	Standard error	P-value	Lower CI 95%	Upper CI 95%
Delay of studies<br>How likely do you think it is that your studies / career will be delayed by the Corona pandemic?	Not likely	Not likely at all	0.6517	1.919	0.3741	0.0815	0.922	3.994
	Partly not likely / partly likely		1.5373	4.652	0.4218	0.0003**	2.035	10.635
	Likely		0.7464	2.109	0.4504	0.0975	0.873	5.100
	Very likely		1.1159	3.052	0.5343	0.0368*	1.071	8.699
Workload of studies<br>How high do you estimate your current level of stress from your studies compared to your usual format?	Significantly less	Significantly higher	-2.0842	0.124	0.7775	0.0073*	0.027	0.571
	Less		-1.6961	0.183	0.4819	0.0004**	0.071	0.472
	Partly less / partly higher		-1.4952	0.224	0.4213	0.0004**	0.098	0.512
	Higher		-0.2677	0.765	0.3968	0.5000	0.352	1.665

* = significant at $\alpha \leq 0.05$ ** = highly significant at $\alpha \leq 0.001$
